# Fruit and Vegetable Quality Assessment via Dielectric Sensing

**DOI:** 10.3390/s150715363

**Published:** 2015-06-29

**Authors:** Dalia El Khaled, Nuria Novas, Jose A. Gazquez, Rosa M. Garcia, Francisco Manzano-Agugliaro

**Affiliations:** 1 Departmentof Engineering, University of Almería, 04120 Almería, Spain; E-Mails: dalia.elkhaled@gmail.com (D.E.K.); nnovas@ual.es (N.N.); jgazquez@ual.es (J.A.G.); rgarciasalvador@ual.es (R.M.G.); 2 BITAL (Research Center on Agricultural and Food Biotechnology), University of Almeria, 04120 Almeria, Spain

**Keywords:** fruits, vegetables, dielectric, quality, sensing, non-destructive

## Abstract

The demand for improved food quality has been accompanied by a technological boost. This fact enhances the possibility of improving the quality of horticultural products, leading towards healthier consumption of fruits and vegetables. A better electrical characterization of the dielectric properties of fruits and vegetables is required for this purpose. Moreover, a focused study of dielectric spectroscopy and advanced dielectric sensing is a highly interesting topic. This review explains the dielectric property basics and classifies the dielectric spectroscopy measurement techniques. It comprehensively and chronologically covers the dielectric experiments explored for fruits and vegetables, along with their appropriate sensing instrumentation, analytical modelling methods and conclusions. An in-depth definition of dielectric spectroscopy and its usefulness in the electric characterization of food materials is presented, along with the various sensor techniques used for dielectric measurements. The collective data are tabulated in a summary of the dielectric findings in horticultural field investigations, which will facilitate more advanced and focused explorations in the future.

## 1. Introduction

Quality is defined by the Spanish Royal Academy as “the property or set of inherent something, you can judge their values” [1]. In 2006, Choi and his co-workers classified fruit quality into internal and external quality factors [2]. Internal factors include the taste, the texture, the value, the aroma, the nutrition and the lack of biotic and abiotic contaminants, whereas the external factors include the presentation, the appearance, the uniformity, the maturity and the freshness of the fruit. Furthermore, the authors stated that although the internal aspects are not noticeable for consumers, they are highly important next to the external aspects that are considered to be the essential purchase decision.

Due to the large consumption increase in the horticulture field, the fresh fruit and vegetable post-harvest sector is dynamic, and the need for high quality produce is rising [3]. Currently, there is a trend to promote the characteristics of vegetable consumption in the diet. It is suggested that the components of vegetables have the capacity to modulate the complex mechanisms involved in maintaining a healthy physiology and reducing the early onset of age dependant diseases, and the demand for agricultural products such as vegetables and fruits is rising [4]. Increasing consumer demand for high-quality fruit has led to the development of optical, acoustic and mechanical sensors that determine its quality. According to Shewfelt [5], the internal characteristics, which are perceivable by the senses of taste, smell, and touch (mouthfeel), are the ones that will determine the decision to repurchase that product. The other characteristics, such as nutritional value, wholesomeness, and safety, cannot readily be determined by consumers because they require measurement, but, if this information is given to the consumer, it will influence acceptability of the product.

In this context, after highlighting the importance of the fruit and vegetable characterization process from harvest to cold storage, and with all of the rapid technological development, mathematical methods and multiplicity of investigations all over the world, there is a huge need for the development of review methods for electrical characterization in the horticultural field [6]. Improved methods for rapidly sensing quality factors of fruits and vegetables, such as moisture content, maturity defects, and blemishes, would be helpful in the harvesting, sorting and packing operations for these commodities; this rapid technique can save labour costs and provide improvement in the uniformity and quality of the products [7].

Moreover, Pliquett [8] stated that the electrical measurement is a simple innocuous tool for material characterization, which should make the determination of electrical properties a highly effective method to enhance the quality of fruits and vegetables. This being said, this information quickly becomes outdated, such that to obtain sustainable and competitive agriculture, it is necessary to use techniques, systems and tools that provide timely monitoring and measurement with reliable information [9].

## 2. Dielectric Characterization of Vegetables and Fruits

### 2.1. Overview

For the past two decades, many researchers have been interested in the study of electrical properties, and numerous experiments were conducted for a large variety of agricultural products (fruits and vegetables). The main factor that affects the dielectric properties of hygroscopic materials is the moisture content; this factor is used as a basis for developing commercial instruments to measure moisture content [10]. As a result of the different mineral substances and organic acids present, along with other components that are susceptible to dissociation, the high electrolytic conductivity of fruits and vegetables is distinguished. Many parameters, such as impedance and permittivity, are quite interesting because dielectric properties are considered to be the most important physical properties associated with radio frequency (RF) and microwave (MW) heating [11].

Due to the interest in the dielectric properties of agricultural materials, which is focused primarily on predicting heating rates that describe these material products when subjected to high frequency heating, the dielectric properties of biological products have become valuable parameters in food engineering and technology [12]. The process of energy absorption through RF or MW energy has been known for a long time and has been widely explored. With the advance of computer modelling tools for RF and MW applications, it is very critical to have data available for the dielectric properties of materials. This issue is noticeable especially in the modern design of heating systems where experiments to meet products have been conducted, and data for the moisture content has been reported for several frequency ranges and temperatures related to the process requirements [13]. Among the factors that are involved in the dielectric properties values, the nature of the material that implies the composition and structure is the most common. Some other factors, such as frequency and temperature, are involved with the maturity stage of the agricultural product. Because MW heating is greatly affected by the presence of water, which is a major absorber of MW energy, the higher the moisture content, the better the heating [14,15,16]. Moreover, ionic components have significant effects on the dielectric properties [17]. Another factor is the density because the amount of mass per unit of volume (density) has a definite effect on the interaction of the electromagnetic field and the involved mass [18]. Another important factor is the storage time of the agricultural products under measure because ripening processes taking place may affect the dielectric properties [19].

**Figure 1:**
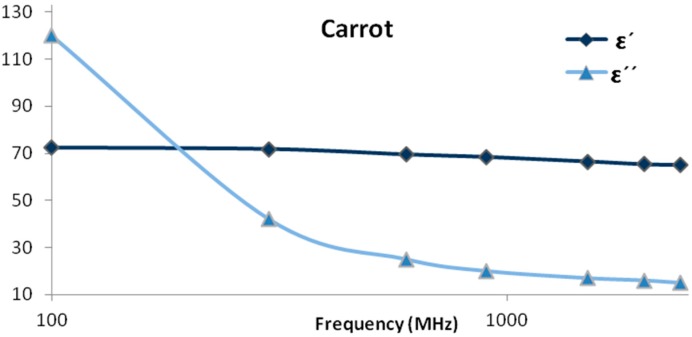
Example of ε′ and ε″ variations with frequency on a logarithmic scale.

The electrical linear properties of tissues and cell suspensions, because of their variation with frequency, are mostly considered to be unusual. These properties include the dielectric constant ε′ and the conductivity, which has been proven to be inversely proportional. To illustrate with an example, a graph is plotted in Figure 1 to show the variations of these parameters with frequency. An interpretation of the parameters behaviour *versus* frequency is to be analyzed in the discussion section to explain the curve patterns. Three distinct major steps accompany the variation of the frequency at low RF and GHz frequencies that are termed as α, β and γ dispersions. Moreover, the dielectric constants reach very high values relative to free space at low frequencies [20]. While the α dispersion remains incomplete for several reasons, the β dispersion is due to the cellular structure of tissues and occurs in the range of 0.1 to 10 MHz. The γ dispersion was noted above 1 GHz for a variety of tissues and protein solutions. In addition to the main dispersion that is due to plasma membranes, the β dispersion possesses additional dispersions on the high frequency side [21].

About the interaction between food and electromagnetic energy at low frequencies, much less is known [22]. At high frequencies, the electric properties of most basic interest are the dielectric properties that affect energy coupling and distribution within the product, which includes the product attenuation constant determining voltage and power penetration depths (Dp) within the product and therefore the temperature at a specific depth [15]. The main advantages of high frequency methods consist of reducing process time, offering more uniform heating patterns and improving product quality in selected applications [23]. By definition, biological materials and their ability to store and dissipate electrical energy are compared to non-ideal capacitors [24]. Energy charging and loss currents related to the material electrical capacitance and resistance are behind these properties, and they are defined as dielectric properties. However, due to the migration of charge carriers, there is a slight difference in the electrical behaviour of a simple resistive-capacitive circuit in conduction and biological materials at high frequency. According to Sarbacher and Edson [25], because the relative magnetic loss of a material is related to the material reluctance and its ability to dissipate magnetic energy, the components of complex permeability, when divided by the permeability of free space, *i.e.*, 1.257 × 10^−6^ H/m, give the components of the complex relative permeability. Alternately, it is necessary to consider the magnetic coupling effects at high frequencies for fruits and vegetables because their relative magnetic permeability is close to unity (magnetic permeability close to that of free space and zero relative magnetic loss) [26].

A material’s ability to attenuate or absorb electrical energy coupled by the material from an electromagnetic field is determined by the real component, which is also the major determinant of energy distribution in homogeneous dielectric materials. The attenuation factor determines the ability of the electric component of the field to penetrate the dielectric and is the reciprocal of the material Dp.

### 2.2. Dielectric Properties

Development of non-destructive and informative sensing techniques to evaluate the properties of living tissues has been a subject of increasing importance for decades [27]. The dielectric properties of food and biological products have become valuable parameters in food engineering and technology [12]. Dielectric spectroscopy is an old experimental tool that has developed dramatically in the last two decades. It currently covers the extraordinary spectral range from 10^−6^ to 10^12^ Hz. Dielectric spectroscopy is a technique used to study the interaction of a material and the applied electric field. It is widely used as a tool for the detection of material aging and fault diagnosis for insulation systems, and hence it has become a popular and powerful research technique [28]. It is based on the phenomena of electrical polarization and electrical conduction in materials [29]. The dielectric property known as complex permittivity is the physical property that describes the interaction between matter and electromagnetic fields, and it is related to the structural and physio-chemical properties, such as water and soluble solids content or water activity, of the material [30]. There are a number of different dielectric polarization mechanisms operating at the molecular or microscopic levels [31]. The analysis of dielectric spectroscopy data gives valuable parameters that characterize the living tissues, such as cell size and shape, the state of the cell membranes and the status of intra- and extracellular media. The dielectric properties of materials that are of interest in most applications can be defined in terms of their relative permittivity. Permittivity is a complex quantity generally used to describe the dielectric properties that influence reflection of electromagnetic waves at interfaces and the attenuation of the wave energy within materials. Based on Maxwell’s equation, the complex dielectric function describes the interaction of electromagnetic waves with matter to reflect the underlying molecular mechanisms [32]. The relative complex permittivity ε* is represented as in Equation (1):

(1) $${\text{ε}}^{*}=\text{ε}´-j\text{ε}´´$$

where ε′ and ε″ are commonly called the dielectric constant and loss factor, respectively, and *j* = √ − 1. The real part, ε′, describes the ability of a material to store energy when it is subjected to an electric field and influences the electric field distribution and the phase of waves travelling through the material. The imaginary part, ε″, influences both energy absorption and attenuation and describes the ability to dissipate energy in response to an applied electric field or various polarization mechanisms that commonly result in heat generation [33]. The amount of thermal energy converted in the food is proportional to the value of the loss factor [13].

In the past, dielectric spectroscopy was characterized by its limitations in frequency, as measurements could usually only be carried out within 4–5 decades of frequency; the dielectric constant and the dielectric loss factor data are presented in sets of points in frequency measured at different temperatures. These measurements show that time superposition holds, within the limited frequency range of the measurement, to obtain the following Debye relaxation function, Equation (2):

(2) $$\text{ε *}={\text{ε}}_{\infty }+\frac{\text{Δε}}{{\left(1+{\left(\text{iωτ}\right)}^{\text{α}}\right)}^{\text{γ}}}$$

where Δε describes the dielectric length, τ is the relaxation time, and α and γ are the quantifications for the symmetric and asymmetric broadening of the relaxation distribution function, respectively. In addition to the molecular dynamic in confining spaces, the scaling of relaxation processes is the major contribution of the broadband dielectric spectroscopy to modern physics [32].

Mechanisms that contribute to the dielectric loss factor include dipole, electronic, ionic and Maxwell-Wagner responses [34], as illustrated by Equation (3):

(3) $$\text{ε}´´=\text{ε}´{´}_{d}+\text{ε}´{´}_{\text{σ}}=\text{ε}´{´}_{d}+\frac{\text{σ}}{\left({\text{ε}}_{0}\text{ω}\right)}$$

where the subscripts d and σ stand for the contributions due to dipole rotation (d) and ionic conduction (σ), respectively; more specifically, σ is the ionic conductivity in S/m, ω is the angular frequency, and ε_0_ is the permittivity of free space or a vacuum (8.854 × 10^−12^). For RF (1 to 50 MHz) and MF (915 to 2450 MHz), σ and d are the predominant loss mechanisms [35]. The power in W/m^3^ dissipated per unit volume in the dielectric can be expressed via Equation (4):

P = E^2^ σ = 55.63*10^−12^ f E^2^ε″ (4)

where *E* represents the rms electric field in V/m.

In this context, we refer to the Kremer statement that although dielectric spectroscopy is old, it is a still-developing experimental technique that has had a strong technological impact and provides variety of novel routes that will open exiting new horizons, such as revealing information about the binding of water in food and other agricultural materials. Thus, further studies should be conducted for the potential applications of dielectric sensing spectroscopy [36].

## 3. Dielectric Sensing Techniques

In this section, the evolution of dielectric properties sensing techniques is described. Dielectric Spectra can be easily obtained using an automated frequency domain spectrometer (FDS) with high precision over a frequency range of 1 Hz to 10 GHz. The measurement techniques appropriate for any particular application depend on the frequency of interest, the nature, both physical and electrical, of the dielectric material to be measured and the degree of accuracy required. In this context, many different types of instruments can be used, and any measurement instrument providing reliable determinations of the required electrical parameters involving the unknown material in the frequency range of interest can be considered.

In the agricultural field, to understand the dielectric behaviour of agricultural products, it was required to boost the measurements over broad frequency ranges and to develop new techniques for efficient collection of permittivity information [37]. The different measurement techniques of the dielectric properties are summarized in Table 1 along with their characteristics.

**Table 1:** Dielectric techniques.

	Brief Description	Recommended Materials	Frequency Range	Advantages	Disadvantages
Parallel plate	Material must be placed between two electrodes to form a capacitor	Material with the ability to be formed as a flat smooth sheet <100 MHz	<100 MHz	Inexpensive, high accuracy	Limited frequency range, sheet sample very thin (<10 mm thick)
Lumped circuit	Sample is a part of the insulator in a lumped circuit	All materials with the exception of gazes	<100 MHz	Liquid and solid materials can be measured	Limited frequency range, not suitable for very low loss materials
Coaxial probe	A coaxial line cut off forming a flat plane boundary in contact with food. A vector analyser is needed to measure the reflection	Liquids and semi-solids	200 MHz–20 GHz, even >100 GHz	Easy to use, non-destructive for some materials, sample preparation is not required	Limited accuracy (±5%). Low loss resolution, large samples and solids must show a flat surface
Transmission line	Brick-shaped sample fills the cross section of an enclosed transmission line, causing an impedance change	Liquids and solids	<100 MHz	More accurate and sensitive than the probe method	Less accuracy than resonators, sample preparation is difficult and time-consuming
Cavity resonator	Sample is introduced in a cavity (a high Q resonant structure), which affects the centre frequency and quality factor of the cavity	Solids	1 MHz–100 GHz	Easy sample preparation, adaptable for a wide range of temperatures	Broadband frequency data are not provided and analysis may be complex
Free space	Antennas are used to direct a MW beam at or through the material. A vector network analyser measures the reflection and transmission coefficients of solids	Solids	MW range	Non-destructive, high temperatures can be used	A large flat, thin, parallel-faced sample and special calibration are required
Time domain spectroscopy	Short pulses of THz radiation within a generation and detection scheme that is sensitive to the effect of both the amplitude and phase of the radiation	Homogeneous	10 MHz–10 GHz.	Fast and high accuracy measurement, small sample	Expensive

### 3.1. Time-Domain Spectroscopy

For some of the studies conducted, time-domain reflectometry and spectroscopy techniques were employed [38]. Time-domain systems generate a fast rise time pulse that is reflected from the sample or transmitted through the sample where the dielectric property information can be extracted by Fourier transform by analysing the waveform. Polymer chemists and physicists have been using time-domain measurements extensively to help in understanding the composition and behaviour of materials [39].

Various techniques over wide ranges of frequency were developed with the presence of suitable equipment for time-domain measurements [40] that have been improved for accuracy [41]. This will enable faster investigations due to the shorter measurement time needed compared with FDS. For measurements below 10 MHz, measuring cells consisting of parallel plate capacitor types are used [42]. However, the residual inductance and capacitance arising from the cell itself and the connecting leads require correction by the measuring cell [43]. At frequencies above 100 MHz, open-ended coaxial probes are suited for measurement with network analysers and time-domain reflectometers. Alternately, for broadband frequency-domain measurements of some products, it was necessary to employ impedance and network analysers with appropriate sample holders and techniques.

### 3.2. Radio-Frequency

For radio frequencies, the material can be modelled electrically at any given frequency as a series or parallel equivalent lumped element circuit. Therefore, if the RF circuit parameters were measured appropriately, the dielectric properties can be determined from proper equations by relating the way in which the permittivity of the material affects those circuit parameters. However, Nelson stated that the challenge of making accurate dielectric properties or permittivity measurements lies in the design of the material sample holder for those measurements and in an adequate modulation of the circuit for reliable calculation of the permittivity from the electrical measurements [44].

The use of the RF measurements in the early stages of the dielectric measurements of agricultural products was very common. Radio frequency measurements relied basically on instruments such as the Q meter [45], impedance bridges [46], and admittance meters [47]. Various bridges and resonant circuits were used as measurement techniques for permittivity or dielectric properties determination in low-frequency, medium-frequency and high-frequency ranges [48]. However, at very low frequencies, invalid measurement data can occur because of electrode polarization; therefore, attention must be paid to the frequency below which polarization affects measurement. This depends on the nature and conductivity of the material being measured [49]. Among the methods used to eliminate the electrode polarization effect are the four-electrode method and the electromagnetic induction method developed with a pair of toroidal coils [50]. With a Q-meter based on a series resonant circuit, a large number of experiments were conducted, and data were obtained in the 1 to 50 MHz range [51]. For higher frequency ranges, coaxial sample holders modelled as a transmission line section with lumped parameters were developed. For the frequency ranging from 50 to 250 MHz, RX meters were used for measurement, whereas admittance meters are used for frequencies ranging from 200 to 500 MHz. By confining samples in a coaxial sample holder, precision bridges were used for audio frequencies from 250 Hz to 20 KHz [52].

A shielded open-circuit coaxial sample holder was created simply by assembling components of the sample holders used in earlier studies with the RX meter and admittance meter to develop a technique of measurement with a frequency range of 100 KHz to 1 GHz with two impedance analysers, proper calibrations and the invariance of the cross ratio technique [53].

The dielectric properties of grains with high frequency bridge measurement from 1 to 200 MHz were determined using a similar shielded open-circuit coaxial sample by Bussey [54] and by Jones and co-workers [55]. Another coaxial sample holder characterized by full two-port scattering parameters was designed and used to provide dielectric properties of grains over the range from 25 to 350 MHz.

### 3.3. Microwave

**Figure 2:**
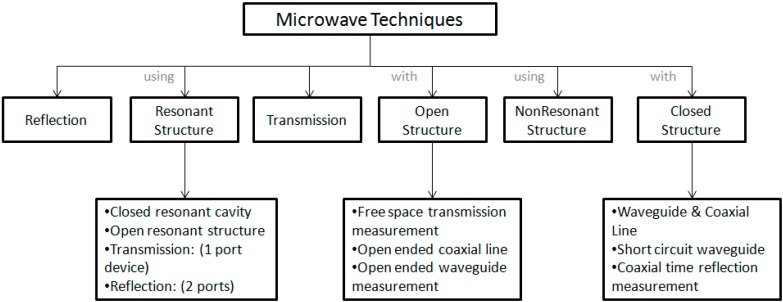
Dielectric property measurement techniques.

Generally, for frequencies that are higher than the one mentioned earlier (1 GHz and above), several MW measurement techniques are available [56]. Other instruments, such as transmission lines, resonant cavities, free space techniques and waveguide systems, were used [57]. MW dielectric properties measurements can be classified as reflection or transmission measurements using resonant or non-resonant systems, with open or closed structures for the sensing of the properties of material samples [58]. Closed-structure methods include waveguide and coaxial-line transmission measurements and short-circuited waveguide or coaxial-line reflection measurements. Nelson described the classification of the MW techniques as follows; Open structure techniques include free-space transmission measurements and open-ended coaxial-line or open-ended waveguide measurements. Resonant structures can include either closed resonant cavities or open resonant structures operated as two-port devices for transmission measurements or as one-port devices for reflection measurements [44]. A schematic diagram of methods to measure dielectric properties is provided in Figure 2.

With the availability of the modern MW network analyser, the efficiency of the methods used to obtain dielectric properties over a wide range of frequencies has increased; these methods cover both time-domain techniques and frequency-domain techniques [59]. To obtain dielectric properties information over wide ranges of frequencies, several frequency-domain systems have been used for measurements on the samples of interest [60]. Moreover, each of these instruments and systems was designed for use over certain ranges of frequency. Table 2 is a graphical presentation of the different types of instrumentation and their appropriate use according to the frequency and material under test.

**Table 2:** Material measurement techniques.

	Illustration	Material Under Test	Frequency	Other Comments
Coaxial Probe	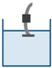	Lossy Material (liquids and semi-solids)	Broadband	Non-destructive
Transmission Line	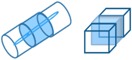	Lossy to low loss material (machineable solids)	Broadband	
Free Space	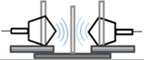	Best for flat sheets, powder high temperature	Broadband	
Resonant Cavity	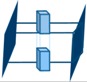	Low loss materials, small samples	Single Frequency	Accurate
Parallel Plate	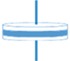	Flat sheets	Low Frequencies	Thin
Inductance measurement	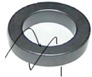	Toroidal structures required		Accurate, simple measurement

### 3.4. Dielectric Sample Holder

An important aspect of the measurement technique is the dielectric sample holder. A suitable method for many materials was provided by the short-circuited technique of Roberts and Hippel [61]. Applying this method, the sample holder is simply a short section of coaxial-line or rectangular or circular termination at the end of the line against which the sample rests. Because the slotted line or slotted section to which the sample holder is connected can be mounted in a vertical orientation, this holder is convenient. In other words, the top surface of the sample can be maintained perpendicular to the axis of wave propagation, which satisfies the measurement requirement.

For fruit and vegetable samples, a rectangular waveguide K-band system was used for measurement [62]. The standing wave ratio (SWR) can be determined by the Roberts and von Hippel [61] method by using the shift of the standing wave node and change in node widths related to the SWR’s. This allows for calculating the sample length and waveguide dimensions ε′ and ε″ with suitable computer programs [63]. However, a network analyser or other instrumentation can provide similar determinations by measurement of the complex reflection coefficient of the empty and filled sample holder [44]. With the development of special calibration methods to eliminate errors caused by unknown reflections in the coaxial line [64], the automatic measurement of dielectric properties has been facilitated by computer control of impedance analysers and network analysers [65]. Moreover, the accuracy and reliability of free space measurements of the MW dielectric properties of agricultural products has always been a priority for developing researchers.

Recently, the technique that is mostly used for measuring dielectric properties of fruits and vegetables and is showing success for convenient broadband permittivity is the open-ended coaxial line. However, some errors might arise with this technique, such as for significant density variations or in case of the presence of air gaps or air bubbles between the end of the coaxial probe and the sample [66]. Construction of a cylindrical cavity might be advantageous for fruit and vegetable applications, especially a cavity with provision for alternate dielectric properties measurements and MW heating of the sample for temperature control [67].

**Figure 3:**
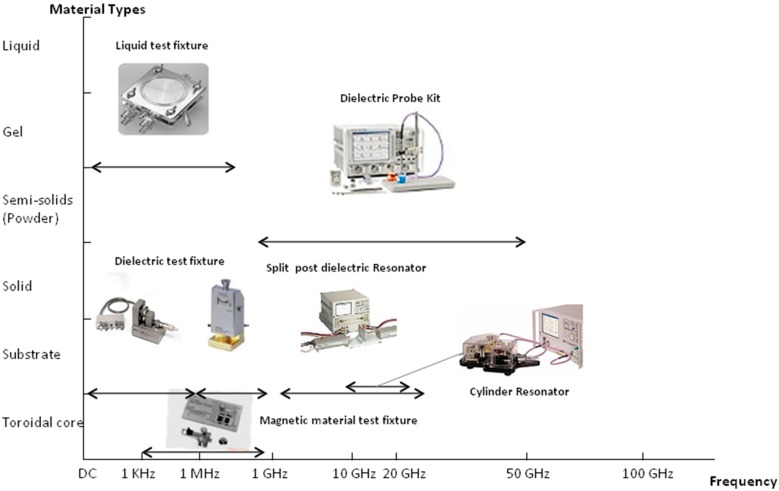
The material measurement fixtures.

To avoid the disturbances resulting from multiple reflections within the sample and between the sample and antennas, a minimal 10 dB attenuation through the sample layer should be maintained for free space permittivity. For extensive studies, it was advised to use the network analysers and impendence analysers, but, for the common limited cases studies, a suitable sample holder can be constructed in available MW laboratories. For cases where the data are required at only one MW frequency or for a limited number of frequencies, a resonant cavity technique is a logical choice [68]; a resonant cavity can be used to measure other permittivity-related characteristics, such as moisture content, mass volume and mixture proportions [69]. Figure 3 is a graphical presentation of the system measurement suite according to the type of material under test and the frequency range adopted for measurement.

### 3.5. Novel Perspective of Dielectric Techniques

In his paper, “dielectric spectroscopy today yesterday and tomorrow”, Kremer developed various novel Perspectives of Dielectric Techniques that are expected to broaden, in his view, the scope of dielectric spectroscopy considerably. First, the principle of hole-burning spectroscopy has been extended to dielectrics and has enabled the answering of questions that cannot be tackled with conventional dielectric spectroscopy [70]. For instance, non-resonant dielectric hole-burning spectroscopy has made it possible to determine experimentally if relaxation processes are homogeneously or heterogeneously broadened. Second, the extraordinary high sensitivity of dielectric spectroscopy enables (frequency-dependent) thermal expansion spectroscopy with high precision [71,72]. Third, dielectric spectroscopy is one of the few spectroscopic techniques that measures directly a molecular fluctuation and has an increasing sensitivity with decreasing thickness, which is a good advantage (this requires refined techniques to deposit electrodes). In this context, a novel approach that allows for avoiding the direct evaporation of metal electrodes has been published [73]. Kremer [32] described an exciting perspective in his view, which is the employment of AFM to carry out dielectric measurement on the very local scale of approximately 50 nm.

## 4. Dielectric Application Data

### 4.1. Apple

Guo and his co-workers [19] measured the dielectric properties of “Fuji”, “Pink Lady” and “Red Rome”, three cultivars of fresh apple *Malus domestica* Borkh, to study their sense of quality. The measurements, executed over 10 weeks in storage at 4 °C, aim to detect whether the dielectric properties are useful for determination of quality factors such as SSC, firmness, moisture content and pH. The experiments were conducted for 51 frequencies on a logarithmic scale from 10 MHz to 1800 MHz for external surface and internal tissue measurements, initially and at 2-week intervals during the 10-week storage period.

During the 10 weeks of storage, the dielectric properties did not change much and instead remained relatively constant. This fact agrees with earlier reported literature regarding the permittivity differences for apple surface and interior tissue measurements [74]. Another point to state is that there are some considerable differences among the three cultivars. The same as for watermelon, the dielectric relaxation phenomenon is also exhibited on the surface measurements that are most likely related to the exocarp structure of the fruit. Taking into consideration the difference in scale, it is noted that the dielectric constant measurements are similar for both the external and internal measurements, but, for the loss factor, the internal tissue measurements show a greater variation than those obtained for the external tissues, especially at lower frequencies. From the obtained results, it is noted that all of the dielectric properties of the three cultivars have the same behaviour with respect to frequency. Additionally, the decline in the dielectric constant is a notable characteristic with increasing frequency. The same phenomena of ionic conduction and dipolar losses at low and high frequencies, respectively, are detected for internal apple measurements as well. As for the surface exterior measurements and considering the frequency range, an overriding dielectric relaxation behaviour that might be due to bound water relaxation is revealed.

To determine the state of fruit maturity through the potential use of dielectric spectroscopy, a study was conducted in 2010 to find relations with apple physiological compounds, such as sugar content and malic acid. A non-destructive control method was presented for the prediction of climacteric fruits maturity. Measurements were taken at a frequency ranging from 500 MHz to 20 GHz. A new good correlation was found between apples’ Thiault Index and a new defined Dielectric Maturity Index. This Dielectric Maturity Index was related to the loss factor at two punctual frequencies (0.5 GHz and dipolar relaxation frequencies) [75].

At the frequency range of 500 MHz to 20 GHz, another dielectric measurement was applied to apples with different sugar contents during ripening. The objective of the study was to determine the optimal time for eating the fruit. Various good new correlations between the newly defined maturity index and the Thiault index were found. Prospective data of some chemical components of apples were presented in the study [76].

To distinguish between diseased and normal fruits, a new theoretical basis for non-destructive inspection and a research method based on electrical properties was developed. The study investigates the change of the law of dielectric properties on the Fuji apple superficial scale. For a frequency ranging from 100 Hz to 3.98 MHz, the dielectric properties of fruits embracing the impedance, reactance, conductance, capacitance and loss coefficient were measured using the Inductance Capacitance Resistance analyser (LCR). The results show that fruit impedance and reactance decrease as frequency increases; the capacitance and dielectric loss coefficient changes are irregular. Only the conductance increases in spiral form for the same storage times. A significant positive correlation exists between impedance and reactance. Moreover, values of capacitance differ between diseased and normal fruits form 100 Hz to 3.98 MHz, which suggests that the quality of apples can be reflected by capacitance to some extent [77].

Using a network analyser and open-ended coaxial line probe, dielectric properties measurements were effected on the external surface, internal tissue and juice of “Fuji” apples during the last two months of tree-ripening. The objective of the study was to determine the relation of permittivity with apple quality by measuring the firmness, SSC, pH, moisture content and electrical conductivity. The observations reveal that the permittivity and electrical conductivity did not show a specific pattern during the ripening period; the moisture content and SSC remained constant, but the firmness and pH increased with maturity. Thus, the study did not find a correlation between permittivity, firmness, moisture content or pH, and the linear relationship between surface permittivity and SSC was poor (<0.2). Calculating the linear regression at 4.5 GHz, the best correlation between the loss tangent of the tissue and SSC and between the dielectric constant of juice and SSC was found to be 0.61 and 0.67, respectively [78].

In the same year, Guo and his co-workers also investigated the dielectric constant and loss factors of apples with skin (skin on), without skin (skin off) and the flesh juice of “Fuji” cultivar for a frequency range from 10 to 4.5 GHz. The firmness, moisture content of the flesh, SSC, pH and direct current conductivity of the juice were under measurement. The results show that with storage time, the pH increases and the firmness decreases, but no pattern was detected for the moisture content, SSC, conductivity or permittivity. With increasing frequency, the depth of penetration in skin-on and skin-off apples and apple juice decreases. Because the correlation between apple permittivity, internal quality and limited Dp was weak, it was difficult to sense the apple internal qualities from the permittivity of skin-on apples, skin-off apples and apple juice. The study concludes that information provided by dielectric properties can be useful in developing thermal treatments for postharvest insect control based on RF, MW energy and non-destructive methods for detecting internal fruit quality as well. Additionally, designing the treatment bed thickness in RF and MW systems and estimating the heating rates of apples samples can be conducted with dielectric properties data. The limited penetrations at frequencies higher than 3 GHz may suggest some unpractical apple quality sensing under the experimental conditions. The study points also to some weak correlations between permittivity and quality indices [79].

Physical information about apples that is closely related to chemical information has been investigated by monitoring apples during aging via electrical impedance spectroscopy as a fast and non-invasive method. The study proposes two different analytical techniques for assessing the apple properties changes. The first method is based on a single measurement at a low frequency range (approximately 100 Hz), while the other is based on an Argand plot. The major changes observed in electric impedance spectra were attributed to changes in the apples’ relative moisture content. Moreover, an equivalent circuit scheme helped to model the apples’ behaviour and derive the apoplastic and simplistic resistances and relaxation times [80].

A two-stage study was conducted in 2013 based on the necessity of finding effective methods to select key features from all other dielectric features to reduce the cost of dielectric signal application in non-destructive detection of fruits and crops. For this, a compact discriminative dielectric feature subset was found at first, and then, based on the first step, a non-destructive apple internal quality estimation system is evaluated. Measurements are executed with nine frequency points ranging from 158 Hz to 3.98 MHz using an LCR tester. Apple samples are graded according to their freshness by weight loss rate (WLR). The apple with WLR equal to 0%, 5%, 10% and 15% are assigned grade one, two, three and four, respectively, whereas grade five apples are those with brown stains. The most discriminative apple internal quality estimation is the compact set of dielectric features. Three classifiers are evaluated at the internal quality estimation stage, the sparse representation classification (SRC), artificial neural network (ANN) and support vector machine (SVM). The results show that fast clustering-based feature subset selection (FAST) selects only four dielectric features with 80% classification rate, while sparse principal component analysis (SPCA) accuracy is mediocre and the performance of the greedy selector is significantly outstanding with classification rates of 91.22% and 95.95%, respectively. The results show that dielectric features are highly relevant to the apple internal quality that can be estimated with a compact set of dielectric features [81].

Another experiment studied the effect of high-voltage pulsed electric field treatment on the dielectric properties of apples using the theory of electromagnetic fields. The results show that when the pulse voltage and the pulse frequency increase, both the equivalent capacitance and equivalent impedance of apples decrease. In fact, the dielectric properties are affected by the high-voltage pulsed electric field parameters [82].

The soluble solid content in “Fuji” apples was predicted by applying the BP network model and support vector regression (SVR) over a frequency range from 10 MHz to 4.5 GHz during 21 weeks of storage. Effects of the full frequency (FF), principal component analysis (PCA) and successive projection algorithm (SPA) prediction models were compared and evaluated. The results show that the PCS-SVR results were better than SPA-BP; the predicted correlation coefficient of PCA-SVR was 0.883, and the root mean square error (RMSE) was 0.552. Moreover, the effect of the PCA-BP was worse than that of PCA-SVR. The RMSE of the established model by SPA was generally smaller than by other methods, and the predicted correlation coefficient of the models established by PCA was generally higher. Based on the frequency spectrum of dielectric parameters, some useful technologies in developing non-destructive sensors for fruits’ soluble acid content were offered as well [83].

Based on the concept that dielectric properties are highly relevant to the internal quality of many fruits and crops but their application is restricted because of their changing frequency, a study was conducted by Li and his co-workers. Its objective was to find an effective dielectric feature selection method to reduce the cost of the dielectric signal application in non-destructive detection of fruits and crops. For this, “Fuji” apples were graded into five levels according to their dielectric features using several methods, such as SPCA, FAST and a ranker method with the attribute evaluator of information gain. The experimental results show that some key dielectric features are sufficient for high classification accuracy [84].

Recently, a study investigated the feasibility of using dielectric spectroscopy as a non-destructive technique to determine the soluble solid contents of fruits. Three varieties of apples were chosen (“Fuji”, “Red Rome” and “Pink Lady”), and their dielectric constants and loss factors were obtained at 51 discrete frequencies from 10 to 1800 MHz using an open-ended coaxial line probe and an impedance analyser. Methods, PCA and the uninformative variables elimination method (UVE-PLS) were applied for the extraction of the dielectric spectra. To establish models that predict the SSC of apples, other methods such as the generalized regression neural network (GRNN), SVM and extreme learning machine (ELM), have served, with calibrated root mean square error and predicted root mean square error of 0.840 and 0.822, respectively. The study also reveals that non-destructive determination of SSC of apples can be done through a combination of dielectric spectra, artificial neural networks and chemometric methods [85].

It is important to mention the existence of other non-destructive methods that have been used for the detection of apples firmness and SSC, such as the biospeckle based on the analysis of laser light variation scattered from the apple sample [86]. Monitoring of quality and ripening of fruits and vegetables, analysis of seeds and assessment of mobility parameters are all applications of the biospeckle technique in the agricultural area [87]. Overall, one can conclude that ionic conduction occurs at low frequencies and dipole relaxation at higher frequencies. A combination of effects including Maxwell-Wagner, bound water, ion related phenomena and molecular cluster are the reason behind the overriding dielectric relaxation of loss factors. For non-destructive surface measurements, the correlations between permittivity and quality indices are not high enough for sensing internal quality. On the other hand, a potential for on-line quality sensing applications may be offered at the tissue and juice levels. All the studies conducted admit that further analysis through radio frequency electromagnetic fields is necessary for a satisfactory quality assessing.

### 4.2. Avocado

In 2003, Nelson [37] conducted measurements of the dielectric properties of the avocado *Persea americana* Miller var. Americana obtained over the temperature range of 5 °C to 95 °C, in the frequency range from 10 MHz to 1.8 GHz. The results show that the dielectric constant dependence with the temperature disappears at some frequency in the range shown; above that frequency, the temperature coefficient for the dielectric constant is negative, and, below that frequency, the temperature coefficient for the dielectric constant is positive. As explained by Nelson, dipole relaxation accounting for most of the energy loss occurs at above that frequency, while ionic conduction is the dominant loss mechanism below that frequency. This critical edge frequency is approximately 100 MHz for the avocado. It is also noted that for lower frequencies, the dielectric constant of the avocado increases in a regular fashion with the temperature increase. The same pattern is observed for the dielectric loss factor, which increases with temperatures at lower frequencies regularly.

Another experiment on avocado products was conducted in 2008 to measure the dielectric properties of food having the potential to be processed using a continuous flow MW heating system at 915 MHz and in the temperature range of 10 to 90 °C. The results show that with the temperature increase, the dielectric constant decreases and the dielectric loss factor increases [88].

Although only poor correlations were observed between moisture content, density and soluble solid content, the research on avocado provided new information that could be useful in understanding the dielectric behaviour for sensing quality.

### 4.3. Carrot

In 2003, Nelson [37] measured the frequency and temperature dependence of the dielectric constant and the dielectric loss factor of the carrot (*Daucus carota* subsp.sativus (Hoffm) Arcang) over the temperature range from 5 °C to 95 °C. The critical edge frequency obtained is approximately 100 MHz for the carrot. It is also noted that for lower frequencies, the dielectric constant of the carrot increases in a regular fashion with the temperature increase. A remarkable peak at 65 °C is noticeable for the carrot tissues before decreasing slightly with the temperature increase to 95 °C. The same pattern is observed for the dielectric loss factor, which increases with temperatures at lower frequencies regularly. As for the frequency increase, both the dielectric constant and loss factor have a decreasing pattern [66].

Recently, an experiment was conducted to find the glass transition temperature from frozen and fried mixtures of carrot fibre plus fructose using dielectric analysis in the range of 200 Hz to 1 MHz. The temperature of the peak derivative assumed to be the transition temperature was found to be frequency-dependent. The moisture was in the range of 2% to 4% when the fructose carrot fibre mixture was spray dried and was free flowing. When the fibre content was higher, no significant difference in stickiness and moisture content was detected. Differential scanning calorimetry of 40%, 50%, 60% and 70% carrot fibre fructose showed transition temperatures of 107 °C, 114.5 °C, 122.9 °C and 130 °C, respectively. The values imply that wall build-up might be avoided in a larger scale dryer [89].

Research concludes that there is point in the frequency range between 10 MHz and 100 MHz where the dielectric constant dependence with the temperature was minimal. The behaviour of the permittivity with temperature can be interpreted by the dominance of ionic conduction and dipole relaxation for frequencies below and above that point respectively.

### 4.4. Coconut Water

Kundu and Gupta applied the same experiment on coconut water. However, because the sample is liquid, the permittivity sensor probe needs to be inserted totally within the liquid to yield accurate measurements. The results show that similarly to other fruits (brinjal, tomato, guava and apple), the real component of permittivity decays in an inverse manner with frequency. The energy storage capability of coconut water was reduced with frequency, and the energy loss appeared to be, at minimum, in the frequency range of approximately 2 GHz [90].

### 4.5. Eggplant (Brinjal)

Under the same conditions of the experiment conducted on tomatoes, Kundu and Gupta measured the permittivity of eggplant (brinjal). The real component of the permittivity decays almost exponentially similarly to tomatoes (to be discussed in section 4.13 of this article) with frequency increase. Over the measured frequency, the dielectric constant is higher at 16 °C than at 25 °C. The relative permittivity of brinjal falls from 36 at 200 MHz to 26 at 8.5 GHz, implying that the energy storage capability decreases with frequency. The value of permittivity is reasonably low due to the presence of less moisture and more air in the brinjal body. The observations of the minimum field energy loss and loss tangent curve indicate that energy storage capability and energy loss within the vegetable vary with ambient temperature [90].

### 4.6. Grape

Using a parallel plate electrode system, the dielectric parameters of red globe grapes were studied as a function of the room temperature and storage time for a frequency range of 50 Hz to 1 MHz. The results show that with the frequency increase, both electric conductance and equivalent parallel capacitance increase, whereas equivalent impedance decreases. At 25 KHz, the loss coefficient reaches its minimum and the quality factor reaches its maximum. With increasing storage time, both the equivalent impedance and quality factor decrease for the same frequency, whereas the equivalent parallel capacitance, the loss coefficient and the electric conductance increase. The study concludes that there is a strong correlation between the electrical characteristic parameters and electric field frequency [91].

### 4.7. Guava

Guava fruit has been measured under the same conditions as tomatoes by Kundu and Gupta [90]. The fruit shows a similar pattern of dielectric constant decrease with frequency increase. The first observation of the study shows that relative permittivity goes up with a temperature increase from 16 °C to 25 °C in GHz frequency scale, implying that the energy storage capability of guava is reduced with frequency. The energy conversion to heat is at its minimum in the frequency band of approximately 1 GHz. The loss within guava, as noted by the loss tangent curves, slightly decreases with the temperature increase from 16 to 25, which goes against Nelson’s reports in 2003 and 2005 [90].

### 4.8. Mango

Sosa-Morales and his co-workers measured the dielectric properties of mangos to understand the interaction between the fruit and electromagnetic energy. Measurements were performed using an open-ended coaxial line probe and an impedance analyser for a frequency ranging from 1 to 1800 MHz, a temperature from 20 °C to 60 °C and 16 days of storage. The results show that the dielectric constant was decreasing with increasing frequency, and the loss factor was decreasing even more so. With increasing temperature, the dielectric constant decreases while the loss factor increases. With storage time, both the dielectric constant and loss factor were decreasing, which is attributed to the reduction in moisture content and increase in pH. The penetration depth (Dp) at which the power drops to 36.8% of its value at the surface of the material was calculated. Obtained results guide that Dp decreases with both temperature and frequency increase, implying that energy penetrates deeper into mangos than MW [92]. This should be expected from the equation developed by Metaxas and Meredith [34] that reflects the relative variation of Dp in function of temperature and frequency.

In 2013, a study was conducted by Yoiyod and Kaririskh [93] on the ripeness monitoring of mangos through a cost-effective remote sensing system. The dielectric properties of the peel and pulp of mango fruit were measured at different maturity stages. A frequency ranging between 6 and 18 GHz is considered to be the most suitable operating frequency. The results show that a significant difference in the reflection coefficient exists between ripe and unripe mangos [93].

### 4.9. Melon

In 2007, Nelson and his co-workers [94] conducted a measurement of honeydew melon dielectric properties to study if a useful correlation exists between the dielectric properties and melon sweetness, as measured by soluble solid content. The measurements show that the mean values of dielectric properties from six probe measurements on internal watermelon tissue reveal a linear relationship for the loss factor between 10 and 500 MHz, which can be interpreted by the dominant influence of ionic conduction in that range [95]. It is essential to mention that a significant difference exists between the measurements executed on internal tissue and those on the surface. Due to the influence of bound water and the complex combination of Maxwell-Wagner and ion-related phenomena, broad dielectric relaxation occurs at the surface measurements, explaining this result difference. Despite the success of relating the dielectric properties of solids in complex plane plots, Nelson [94] stated that the search for correlations for predicting melon sweetness from the dielectric properties was not successful so far.

In 2011, Nelson and Trabelsi [96] re-examined the earlier dielectric spectroscopy measurements of honeydew melons and watermelons from 10 MHz to 20 GHz. The objective of the study was to find useful correlations with SSC (sweetness) for non-destructive sensing of melon quality. The study could not reveal any new information regarding the melon quality through dielectric properties. The internal edible tissues useful for quality sensing were attenuated by RF signals. However, better coefficients of variations were obtained at higher frequencies.

### 4.10. Orange

Nelson [37] measured the dielectric properties for the navel orange (*Citrus aurantium* sbsp. Bergamia). The frequency at which the temperature coefficient reverses its sign, known as the frequency of zero temperature dependence, is noted to be at approximately 50 MHz [36]. A phenomena occurs at lower frequencies where large ionic conduction masks the biological cell constituents and the dielectric relaxation forms of bound water [97]. This explains why the reversal of the sign of the temperature coefficient is evident only for the dielectric constant and not for the case of the dielectric loss factor. However, for high frequencies, a shift in the relaxation frequency for liquid water to higher frequencies occurs with temperature increase, resulting in the reversal of the temperature coefficient for the loss factor at the high end of the frequency range. The relaxation frequency of liquid water is 10.7 GHz at 50 °C; thus, the associated dispersion is evident above 1 GHz. The polarization at lower frequencies in cellular structures is mainly due to the increased ionic diffusion and ionic conductivity processes, which cause the high values of the dielectric constant at low frequencies [98]. As evidenced by the data, when the temperature increases, this dispersion might also shift to higher frequencies. Nelson also concluded that the ionic conductivity phenomena which increases with frequency falls in keeping the convention of the Kramers-Kronig relations. This critical edge frequency is approximately 40 MHz for the navel orange. It is also noted that for lower frequencies, the dielectric constant of the orange increases in a regular fashion with temperature increases. The same pattern is observed for the dielectric loss factor, which increases with the temperature at lower frequencies regularly.

### 4.11. Peach

In 1995, Nelson [7] measured the dielectric properties of tree-ripened peaches of different maturities. Among the three categories of peaches included in the study, the “Dixired” were the first to mature, followed by “Redheaven” and “Windblo.” An attempt to narrow the maturity range was taken via non-destructive measurements that have been associated with maturity in experimental work [99], taking into account both the blush and the ground sides of the peach fruit to accommodate any differences that might be noted on the same fruit (5% accuracy as specified by Helwett-Packard).

The results of their experiments prove that the dielectric constant of the peaches at different maturities are diverging at the lower end of the frequency range; therefore, it seems interesting to explore the behaviour of the constants for other fruits and vegetables at a lower range of frequencies (below that point). Generally, standard deviations for the 15 permittivity measurements on tissue samples of the five fruits were less than 2% for the dielectric constant and less than 10% of the loss factor. It is also noticeable that maturity is accompanied by a slight increase in the dielectric constant at 200 MHz; however, the dielectric loss factor shows only a slight dependence on the stage of maturity. At a higher frequency, e.g., 10 GHz, the dielectric loss factor tends to increase with maturity, whereas the dielectric constant has no consistent trend across the samples. In sensing the stage of maturity, Nelson and his co-workers suggested from the data obtained that values of ε′ at lower frequencies and values of ε″ at higher frequencies should be useful. Therefore, a permittivity maturity index was developed, as shown in Equation (5):

(5) $$M𝑝=\frac{{\left(\text{ε´}\right)}_{0.2}+{\left(\text{ε´´}\right)}_{10}}{100}$$

A second maturity index based on permittivity, utilizing both real and imaginary components, was formulated as Equation (6):

(6) $$Mltr=\frac{{\left(\text{tanδ}\right)}_{10}}{{\left(\text{tanδ}\right)}_{0.2}}$$

This new ratio takes into account the fact that ε′ shows more variation with maturity at 0.2 GHz and ε″ shows more maturity dependence at 10 GHz. Thus, their product should be useful. To cover both real and imaginary components of the permittivity, the ratios ε′/ε″ at 0.2 GHz and ε′/ε″ at 10 GHz would be relevant with the use of the loss tangent tan δ (defined as the ratio of ε′/ε″). Based on the experiments, the dielectric constant and loss factor, which are the real and imaginary components of the complex permittivity, respectively, are showing a significant variation with frequency over the range from 200 MHz to 20 GHz. Because the values appear to be diverging with respect to maturity indices, more effort should be required in this area for more reliable maturity indices in the non-destructive sensing of maturity for peaches. This being said, Nelson’s experiment could reach two maturity indices development but could not reveal any new information behind the data variations of ε′ and ε″.

In 2007, an experiment based on electrical properties measurements was conducted to investigate the electrical and physiological properties of peaches. The study was seeking a better understanding of the electrical properties of post-harvest fruits and a deeper exploration of new quality sensing methods. With peach aging, the loss tangent was observed to decrease and the relative dielectric constant to vary with the cosine law, roughly. At the peak of respiration, the maximum relative dielectric constant appeared [100].

Also in 2007, the internal quality of just-harvested early- and mid-season peaches was studied using MW spectroscopy. Establishing the feasibility of MW measurements for the firmness and sugar estimation of the peaches was the objective of the study. Using a contact coaxial probe from 1 to 20 GHz, the return loss (LR), a MW parameter, was measured simultaneously with reference parameters such as firmness, acidity, sugar content and optical reflectance. Because the MW response changes significantly with the treated samples, the results show that the dielectric response in the peach fruits is affected highly by moisture content and temperature. In fact, when fruits are submerged in water for 1 h (moist) and the temperature is cold (1 °C), the LR increases 50%–100% compared with fruits at ambient temperatures (20 °C). Return loss values obtained at different measured frequencies (1, 7, 9.9 and 19 GHz) show high correlations for the same fruit. However, low correlation is observed with fruit firmness, which is not consistent enough to be applied for fruit inspection and sorting purposes. The study demonstrates a low repeatability of the LR and concludes that the most significant independent variable for estimating peach firmness is the reflectance at 680 nm. Moreover, the variance that is the coefficient of determination of the firmness models reaches approximately 50% to 60%, with lower values for sugar estimation models [101].

In 2010, peaches with different maturities were selected to explore their dielectric properties and determine their internal quality. Experiments were held at 25 °C using coaxial open-ended probe technologies over a frequency range from 10 to 4.5 GHz. Measurements of the moisture content of peach pulp, soluble solid contents (SSC), pH value and electrical conductivity show that the dielectric constants of the pulp and juice decreased with increasing frequency, while the loss factors change with “V” type. The major loss mechanisms at lower and higher frequencies were the ionic conduction and the dipolar polarization. Moreover, the relationship between permittivity and soluble content, pH value and moisture content was non-linear [102].

In order to understand the interaction between the electromagnetic fields and to design treatment beds in industrial applications, an experiment investing the peaches dielectric properties was conducted recently. The study was based on the fact that MW and radio frequency methods hold potential for postharvest thermal disinfestations to replace chemical fumigation. Using an impedance analyser, the dielectric properties of the peaches were determined between 10 and 1800 MHz over a temperature ranging from 20 to 60 °C. The results show that the dielectric constant varies between 60 and 75, accounting for an 8% to 10% change due to the temperature effect. When temperature increased from 20 to 60 °C, the loss factor decreased linearly with frequency on the log scale by approximately 110%, and the loss factor ratio was 1.66 at 20 °C. The penetrating depth decreased with increasing frequency [103].

### 4.12. Potato

Dielectric properties of potatoes were measured with a system consisting mainly of an impedance analyser, high temperature coaxial cable and a metal sample holder. When increasing the temperature from −20 °C to 0 °C, both the dielectric constant and loss factor rapidly increase. From 0 °C to 100 °C, the dielectric constant decreases linearly, while the loss factor decreases first and then linearly increases [104]. With a frequency increase, both the dielectric constant and loss factor have a declining pattern [66]. Results show that the thermal properties found are critical input parameters for a microwave heat transfer model. Thus, authors suggested implementing this model to be used by food scientists in developing novel products that minimize non-uniform heating during cooking in domestic microwave oven.

### 4.13. Tomato

De los Reyes [30] measured the dielectric properties of tomatoes at three locations: the centre of the pericarp, the locular cavity and the skin. However, the measurements at the skin were not considered to be successful because of the non-permeability of the cereus skin to the mass transfer or the bad connection between the probe and the tomato skin. The mean values of six replicates of tomato samples were measured by the authors from 200 MHz to 20 GHz at 21 °C. It is remarkable that large drops in the Dp may damage the fruits due to elevated power absorption.

Over a frequency ranging from 30 MHz to 3000 MHz, a study was executed in 2013 to determine the tomatoes dielectric properties. Open-ended coaxial probe technique was used. The effects of NaCl and CaCl_2_ were investigated on three tomato tissues separately: the pericarp (including the skin), the locular tissue (includes the seeds) and the placental tissue. The results show that with increasing frequency and salt addition, the loss factors of the three tomato tissues decrease. However, with increasing temperature, the loss factors decrease initially but then increase at 915 MHz. The ionic conductivity is the main reason behind the differences in the loss factors of the three tomato tissues [105].

Recently, Kundu and Gupta measured the dielectric properties of tomato fruits for a frequency range from 0.5 GHz to 5.5 GHz at two different temperatures, 16 °C and 25 °C, using the Agilent 85070E Dielectric Probe Kit and Network analyser. The objective is to note the switch in dielectric constant and dielectric loss factor. Although the permittivity curve of tomatoes is higher at 16 °C than at 25 °C, results show that the dielectric constant decays exponentially with frequency increase at both temperatures. The relative permittivity of tomatoes falls from 63 at 200 MHz to 40 at 8.5 GHz. This implies that the energy storage capability of tomatoes is reduced with frequency, but a high permittivity is always expected due to high moisture content. Moreover, the loss tangent is minimal at approximately 1 GHz to 2 GHz, and the dielectric loss factor increases. This implies that the energy conversion is also minimal in the frequency range of 1 GHz to 2 GHz [100].

## 5. Discussion

Sensing food quality through dielectric spectroscopy reveals some very impressing results and opens the horizon for better food consumption in a society where quality is the key for food industries success. Thus, the focus was to build a solid literature review of the various species of fruits and vegetables that have been measured for their dielectric properties. The topic might seem straightforward but experiments varied much in their hypotheses and circumstances. As the paper shows, for the same type of fruit “apple”, authors might come out with different results according to the nature of experiments conducted. For example, apples in each experiment were tested for different quality indices (firmness, ripening, soluble solid content, sugar content, moisture content) and the factor under scope was considered by changing different environmental parameters (temperature, frequency, storage period). Moreover, the quality index varies from one fruit to another which multiplies the possibilities of experiments and makes it an unlimited field. This explains the variation in the experiments that were never unified for only one aim. For the same type of fruit, quality estimation could be measured from different angles seeking improvement.

**Figure 4:**
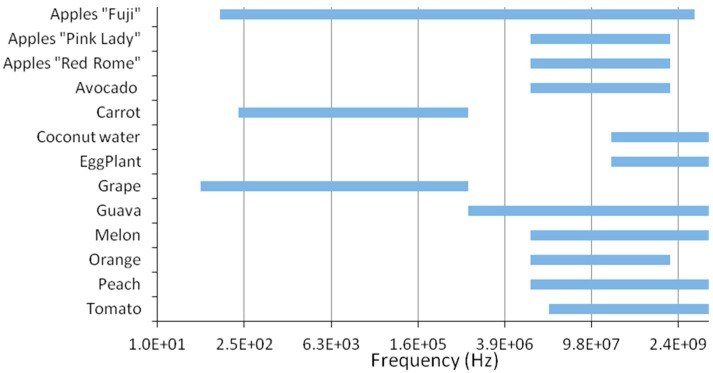
Frequency Ranges applied for fruit and vegetable measurements.

Regarding the frequency range, it is not consistent within the different apple experiments and for the other fruits as well. Some were conducted at low frequency range; however recent ones have been applied at higher frequency ranges. Recently, high-frequency sensing measurement has been approachable with the availability of advanced technology instruments. It is important to note that a higher frequency requires higher budget cost which might be the obstacle for many of the researches. Throughout the paper, it was shown that higher frequency lead to more focused results. The various frequency ranges executed on the different species of vegetables and fruits are presented graphically in Figure 4.

After comparing the results of several experiments conducted on fruits and vegetables, only the dielectric constant measurements of apple fruit by Kundu and Gupta were similar and repeated in most of the studies and verified Nelson’s work in 2005, 2007 and 2008. In most of the vegetables rich in water, the real component of the permittivity shows reasonably high results due to the moisture content with the exception of brinjal, where relative permittivity was lower due to lower moisture. Thus, the results demonstrate the fact that higher permittivity is expected in fruits and vegetables with higher moisture content [100]. The loss tangent curves show that the loss is at a minimum at approximately 1 GHz to 2 GHz for most of the measured samples, which implies that dielectric loss is minimum at that frequency band and that the RF field provides an optimum angular velocity for the polar particles in those samples. In other words, the friction becomes nominal to reduce the heat generation within vegetables and fruits. For the apple, tomato, eggplant and coconut water, it is remarked that the relative permittivity decreases and the loss tangent increases with increases in temperature (from 16 to 25). Only the guava fruit is an exception. Both observations of Nelson, in 2003 and 2005, are in concordance with Kundu and Gupta. It may be that maturity levels of guavas were not exactly the same, which resulted in different temperature dependence for the dielectric loss and loss curve for guava.

For apples [19], the dielectric measurements applied on different parts of the fruits did not show similar results; the same was true for the surface and internal measurements that were considerably different. Lower dielectric constants were observed at the surface of apples, and much lower dielectric loss factors at the surface than at the internal tissues with increasing frequency, regularly pointing to the dominance of ionic conduction at lower frequencies and dipolar relaxations at high frequencies. Additionally, the dielectric properties remained relatively constant throughout the storage duration of 10 weeks. To sense apples’ quality factors, wider frequency range analysis must be necessary through RF electromagnetic fields. Moreover, the fact that extracellular resistance is lower than intracellular resistance implies a disproportionate relation between resistance and permittivity constants. This statement is verified by Keller and Licastro, who proved that the high resistivity was associated with lower water content and that a high dielectric constant is associated with high water content [106]. In practice, this would mean that the surfaces of the apples having high resistivity and lower permittivity is poor in water content, whereas the internal part of the apple showing lower intracellular resistance and higher permittivity values is rich in water. One can conclude that permittivity and water content are positively correlated; then, because resistivity and water content are negatively correlated, resistivity and permittivity are thus negatively correlated. Table 3 shows a summary of the different dielectric parameter variations with various experimental factors; a conclusion on the permittivity pattern is accompanied.

**Table 3:** Parameter Variation overview (Δ & ∇ for parameter increases and decreases, respectively).

Fruit/Vegetable	Frequency			Temperature			Storage Time			Conclusions
	ε′	ε″	Others	ε′	ε″	Others	ε′	ε″	Others	
Apple	∇		Impedance ∇ Reactance ∇				No pattern	No pattern	Conductance Δ Firmness Δ pH Δ	Linear decrease with frequency
Avocado				At low freq. Δ At high freq. ∇	At low freq. Δ					Inflection point at Critical edge freq. 100 MHz
Carrot	∇	∇								Inflection point at Critical edge freq. 100 MHz
Coconut	∇									Linear decrease with frequency
Eggplant	∇									Linear decrease with frequency
**Grape**			Conductance Δ Capacitance Δ Impedance ∇					Δ	Equ. Capacitance ∇ Equ. Parallel capacitance Δ conductance Δ	Linear increase with storage time
**Guava**	∇		Energy storage capability ∇	Relative permittivity Δ	Relative permittivity Δ					Linear decrease with frequency
**Mango**	∇	∇	Dp ∇	∇	Δ	Dp ∇	∇	∇		Linear decrease with frequency
**Melon**										Frequency linear relationship between 10 and 500 MHz
**Orange**				At low freq.Δ	At low freq.Δ	Dispersion shift to higher freq.				Temperature linear increase below 50 MHz
**Peach**	∇ & Std. < 2%	V type & Std. < 10%	Dp Δ High correlation of:LR & freq. and LR & fruit firmness		linear ∇					Frequency and temperature linear decrease
**Potato**	∇	∇		From −20 °C to 0 °C Δ From 0 °C to 100 °C ∇	From −20 °C to 0 °C Δ From 0 °C to 100 °C Δ then ∇					Frequency linear decrease, varying temperature pattern
**Tomato**	Exponential ∇	∇			∇ then Δ at 915 MHz					Exponential relationship with frequency

The experiments shown earlier contribute in framing the permittivity constant variations and determining its major influencing factors. The composition of the fruit, especially the water content, has a positive correlation with the dielectric constant and dielectric loss factor. Indeed, the water is the major absorber of energy in foods; thus, the higher the moisture content, the better is the heating [107]. Therefore, dehydrated fruits show a decrease in their permittivity level compared to other fruits rich in water.

Regarding ionic components, it was observed that salts result in augmenting the dielectric loss factor in mashed potatoes, while the dielectric constant was not affected [17]. Because the physical structure considerably affects the dielectric properties, density is another important factor observed to change with measurements, e.g., the lower the density, the lower is the permittivity. In fact, Guo suggested some simple relations to estimate the chickpea flour dielectric properties from the density and vice versa where it was concluded that the dielectric properties increased with the increase in density and moisture content (from 1.2698 to 1.321 g/cm^3^and 1.9 to 20.9%, respectively) [108].

In regard to temperature, this factor has a strong yet complex influence over the dielectric properties but depends initially on the food composition and moisture content [13]. It can be stated generally that at low frequencies, due to the ionic conductance, the loss factor increases with the temperature increase [17]. In contrast, at high frequencies, due to free water dispersion, the dielectric constant decreases with increasing temperature [109]. These theories have been verified through the experiments. As was discussed for the avocado fruit, the temperature coefficient is positive below 100 MHz, where both the dielectric constant and dielectric loss factor were increasing with temperature; in contrast, the temperature coefficient was negative for frequencies higher than 100 MHz. The pattern was also observed for the navel orange measurements, where the loss factor was increasing with temperature at lower frequencies in a regular fashion, and a massive explanation of the ionic diffusion was a subject of interpretation. Similarly, the peak temperature of carrots reached 65 °C.

**Figure 5:**
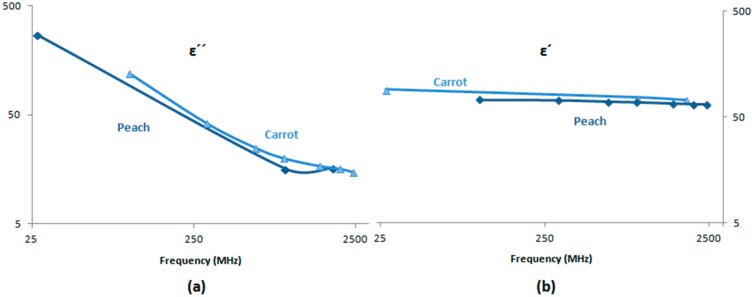
(**a**) Loss factor behaviour *versus* frequency for carrots and peaches on a logarithmic scale; (**b**) Dielectric Constant behaviour *versus* frequency for carrots and peaches on a logarithmic scale.

In regard to frequency, most lossy materials, *i.e.*, materials that absorb and loose energy from RF or MW heating, have dielectric properties that vary considerably with the frequency variation. The imposed electric field and its orientation influence the polarization of molecules, resulting in the dependence of dielectric properties and frequency [48]. At MW frequencies, both σ and d (of free water) play a major role, whereas only σ is dominant at lower frequencies (<200 MHz). This phenomenon was observed for the avocado fruit, for which Nelson attributed the energy loss at high frequency to the dipole relaxation and the ionic conduction at low frequencies [37]. Figure 5 shows the loss factor and dielectric constant behaviour of carrots [66] and peaches [110]. The same pattern is concluded after the navel orange measurement conduction as well. While the dielectric constant is always decreasing with the frequency increase, the loss factor patterns prove to have either a declining curve (carrots) or a V-type curve with a point of inflection (peaches). This critical frequency point identifies each product and characterizes its behaviour. Thus, fruits and vegetables cannot be distinguished according to the dielectric constant that has similar behaviour, but the loss factor can be characterizing because it differs from one product to another.

Concerning the peel of the fruit, an experiment tackling the disinfestation of fruits adopted the radio frequency heating as a fast and volumetric method to overcome the problems of conventional heating. The results of the measurements conducted via open-ended coaxial probe methods show that the RF heating behaviour of the fruits is greatly influenced by dissimilarity in peel and pulp dielectric properties. For apples, peeled oranges and grapefruits, core heating was prominent, whereas it was subsurface or peripheral heating for whole oranges, grapefruits and avocados (fruits with thicker peels). This is expected due to the difference in dielectric and physical properties of the pulp and peel [110].

Last but not least is the storage time factor, which directly affects a large number of investigated fruits. Because fruit and vegetable ripening occurs during storage time, storage time effect studies are one of the main goals of the dielectric properties experiments conducted so far. Storage time, being considered as a major quality factor, was significant in the apple study; dielectric constants and loss factors of the apples remained constant during the 14 weeks of storage, which revealed the importance of further research at wider frequency ranges. The case of apples is quite different than that of mangos, whose dielectric properties decrease with storage time. The phenomena of decreased permittivity in consideration of storage time can be attributed to the increase in pH and decrease in moisture content. The effect of the storage time on the dielectric properties differs from that of the electrical conductivity, which increases with temperature during the storage time [107].

The dielectric studies reached various good conclusions regarding the development of maturity indices. However, further investigation at higher ranges of frequency are always needed for better accuracy, especially in the areas where dielectric properties data do not show significant variations upon which the author can build an effective hypothesis. Moreover, dielectric measurements are distinguished by not being destructive, at least for the experiments shown in this review. The destructivity of such method could be reached at very high frequencies, causing damages to the fruit membranes. In addition, for fruits or vegetables where the membrane is thick, damage is less likely than for fruits with very thin membranes, or by performing external or internal measurements. The importance of these measurements is not only for the detection of the typical dielectric properties but also to provide information on the dependence on variables, such as the alternating field applied, the moisture content and the temperature of the products [95]. In many of the cases, measurements for the same type of agricultural product were duplicated over time with more improved measurements, using several frequency domain measurement systems [60] starting with the RF measurements, the MW measurements, time-domain reflectometry and open-ended coaxial line probes. Investigations of dielectric heating treatments of materials at different frequency ranges may show a higher level of mortality. Some new measurements have been taken to establish a database for a few different fruits and vegetables [37], but comparisons of tissue from fruits or vegetables of different maturities have not yet been tested.

All these indices contribute positively to the hypothesis of valid dielectric characterization of fruits and vegetables. Each of the horticultural products has its appropriate comportment for ε′ and ε″. It was sufficient for some products to be characterized through one constant behaviour while others need both and some others are not yet determined. It is a three dimensional system of frequency temperature and dielectric parameters.

To summarize, measuring permittivity and loss parameters of different agricultural products can be useful in employing RF dielectric heating. According to the dielectric materials to be measured, the choice of measurement techniques, equipment and sample holder design are performed; additionally, the frequency range of interest is an important factor as well [44]. In addition to dielectric spectroscopy, other sensors for quality detection are being used, such as E-noses sensors that use metal oxide conductors. Theses sensors have been applied to a wide range of food and beverages [111]. Once the measurement systems and techniques have been developed and their reliability has been verified, the recently developed instrumentations and techniques make the assembly of dielectric properties information or other techniques much more efficient [95].

## 6. Conclusions

Sensing food quality through dielectric spectroscopy reveals some very impressing results and opens the horizon for better food consumption in a society where quality is the key for food industries success. The paper aims to enlighten the importance of this sensing technique, describing the availability of its instruments, and presenting a state of art of the topic. So, this review summarizes the potential of dielectric properties to investigate functional relationships with temperature, frequency, MW, soluble solid content, moisture content and other processing parameters with sensing the fruit maturity and firmness, knowing that materials undergo physiological changes that will affect the electric property measurements. The application section summarizes from the recent practical research conducted in 13 fruits or vegetables: apple, avocado, carrot, coconut, eggplant, grape, guava, mango, melon, orange, peach, potato, and tomato. All of the obtained conclusions lead to a strong correlation of the dielectric properties with the quality fruit factors at various levels. They aim for a more reliable fruit characterization through the development of rigid permittivity-based indices or solid resistance-based indices in non-destructive environments. The multiple experiences were executed using a variety of instruments and techniques, which implies that a huge market is available for such systems. However, the difficulties faced in many MW applications should be directed towards better designs that are adaptable to the structural changes that might occur during heating or other physical interactions. This review collects a variety of experiments that have been tested, but further research experiments should be conducted for better oriented conclusions and more precise hypotheses.

## Acronyms

**Nomenclature**	**Definition**
α	Quantification of the symmetric broadening of the relaxation distribution
τ	Relaxation time
σ	Ionic conductivity
γ	Quantification of the asymmetric broadening of the relaxation distribution
ω	Angular frequency
Δ	Increase
∇	Decrease
d	Dipole rotation
*E*	Rms electric field
ε*	Complex relative permittivity
ε′	Dielectric constant
ε″	Loss factor
Δε	Dielectric length
α dispersion	Alpha dispersion
β dispersion	Beta dispersion
λ dispersion	Gamma dispersion
*f*	Frequency
L_R_	Return loss
M_ltr_	Permittivity maturity index
M_p_	Permittivity maturity index
tan δ	Loss tangent
BP	BP network model
Dp	Penetration depth
ELM	Extreme Learning Machine
FAST	Fast clustering based feature subset selection
FDS	Frequency domain spectrometer
FF	Full Frequency
GRNN	Generalized Regression Neural Network
LCR	Inductance Capacitance Resistance analyser
MW	Microwave
P	Power dissipated
PCA	Principal Component Analysis
PCA-BP	Principal Component Analysis using the BP model
PCA-SVR	Principal Component Analysis using the SVR model
PCA-SVR	Principal Component Analysis using the SVR model
RF	Radio Frequency
RMSE	Root Mean Square Error
SPA	Successive Projection Algorithm
SPA-BP	Successive Projection Algorithm using the BP model
SPCA	Sparse Principal Component Analysis
SRC	Sparse representation classification
SSC	Soluble Solid Content
SVM	Support Vector Machine
SVR	Support Vector Regression
SWR	Standing Wave Ratio
UVE-PLS	Uninformative Variation Eliminations
WLR	Weight Loss Rate
